# 595 Capability Challenges in a Verified Burn Center During Disaster

**DOI:** 10.1093/jbcr/iraf019.224

**Published:** 2025-04-01

**Authors:** Lindsay Desantis, Laurin Proctor, Samantha Allbritton, Lily Daniali, Ryan Endress, Wojciech Przylecki, Benson Pulikkottil

**Affiliations:** HCA HealthONE Swedish; HCA HealthONE Swedish; HCA HealthONE Swedish; HCA HealthONE Swedish; HCA HealthONE Swedish; HCA HealthONE Swedish; HCA HealthONE Swedish

## Abstract

**Introduction:**

The existing literature on the capability challenges faced by verified burn centers in the United States is notably sparse. The response of these centers during a Burn Mass Casualty Incident (BMCI) is contingent upon the resources and personnel available. This case study explores the operational capability of a verified burn center in the context of a sudden influx of critically injured burn patients following an oil rig explosion.

**Methods:**

Following an oil rig explosion, three patients with greater than 20% total body surface area (TBSA) burns were simultaneously admitted to a verified burn center. Upon notification of incoming patients, the burn leadership team initiated a structured operational response, fostering collaboration among clinical staff and hospital leadership while addressing anticipated challenges. Due to a limited number of burn-trained nurses, a team-based nursing model was implemented, allowing one burn critical care nurse to coordinate care for all three patients, supported by non-burn critical care nurses. At the time of the incident in the eight-bed dedicated burn intensive care unit (BICU), three patients with severe burns were actively receiving acute critical care. The facility incident command assembled in anticipation of capability challenges to determine resource assessment, preparation, response, triage, and recovery.

**Results:**

All patients survived without major complications, with no cases of hypothermia or over-resuscitation. Proactive communication facilitated effective resource allocation, despite statewide shortages of colloid products and burn-specific dressings. During the admission and triage phase of care, burn center leadership determined that one additional critical care burn patient admission would lead to an activation of a Stage 1 Burn Disaster for the facility due to the projected hyperextension of resources.

**Conclusions:**

The capability of a burn center can be significantly strained by the admission of a small number of critically injured burn patients. A structured operational response is imperative for enhancing patient survivability in a facility-based BMCI scenario. Clear identification and communication of resource limitations to incident command are critical. It is essential that burn centers routinely practice and update their Burn MCI response protocols.

**Applicability of Research to Practice:**

This study contributes to the limited literature addressing real-time capability challenges in burn centers, offering insights for optimizing resource management and disaster preparedness.

**Funding for the Study:**

N/A

